# Inhibition of USP14 Ameliorates Sepsis‐Induced Acute Kidney Injury by Suppressing NLRP3‐Mediated Pyroptosis

**DOI:** 10.1002/iid3.70597

**Published:** 2026-11-10

**Authors:** Jini Zheng, Ming Zheng, Dan Zhu, Qian Zhao, Lei Zheng, Yangyang Ye, Xiaoqiong Huang, Wanglong Chen

**Affiliations:** ^1^ Department of ICU Affiliated Yueqing Hospital of Wenzhou Medical University Wenzhou China; ^2^ Department of Orthopedics Affiliated Yueqing Hospital of Wenzhou Medical University Wenzhou China

**Keywords:** NLRP3, pyroptosis, sepsis‐associated acute renal injury, USP14

## Abstract

**Purpose:**

Sepsis‐associated acute kidney injury (SA‐AKI) presents a considerable clinical challenge, marked by high mortality rates and few treatment alternatives. The objective of this research was to investigate the involvement of ubiquitin‐specific protease 14 (USP14) in the development of SA‐AKI.

**Methods:**

Cecal ligation and puncture (CLP) surgery models in male C57BL/6 mice, along with lipopolysaccharide (LPS)‐induced HK‐2 cells, were utilized to simulate SA‐AKI in vivo and in vitro. Renal function was assessed by measuring serum concentrations of creatinine and blood urea nitrogen (BUN). Renal injury was assessed through histopathological analysis. Pyroptosis was measured using enzyme‐linked immunosorbent assay, flow cytometry, and Western blotting techniques. The relationship between USP14 and NLR family pyrin domain containing 3 (NLRP3) was investigated using co‐immunoprecipitation (Co‐IP) and ubiquitination assays.

**Results:**

USP14 expression was upregulated in the kidneys of CLP mice, which correlated with increased the serum creatinine and BUN levels, as well as renal histopathological distruction. The knockdown of USP14 improved renal function and reduced renal tubular injury. Furthermore, USP14 knockdown suppressed the expression of pro‐inflammatory cytokines and decreased NLRP3, N‐terminus of Gasdermin D (GSDMD‐N), as well as cleaved Caspase1, indicating a reduction in pyroptosis. Co‐IP assays demonstrated a physical interaction between USP14 and NLRP3, while USP14 knockdown increased NLRP3 ubiquitination, suggesting that USP14 negatively regulates NLRP3 stability. *In vitro*, USP14 knockdown in LPS‐stimulated HK‐2 cells resulted in reduced inflammation and pyroptosis.

**Conclusion:**

USP14 deubiquitinates NLRP3, promoting NLRP3‐mediated pyroptosis, which contributes to renal injury. Inhibition of USP14 may serve as a potential therapy for mitigating SA‐AKI.

## Highlights

- The knockdown of USP14 alleviated SA‐AKI.
- USP14 promoted pyroptosis in SA‐AKI.
- USP14 induced pyroptosis in renal tubular cells through the deubiquitination of NLRP3.

**Abbreviations**
ANOVA: analysis of variance
ASC: apoptosis‐associated speck‐like protein containing a CARD
BCA: bicinchoninic acid
BUN: blood urea nitrogen
CCK‐8: cell counting kit‐8
CLP: cecal ligation and puncture
Co‐IP: co‐immunoprecipitation
DAMPs: damage‐associated molecular patterns
ELISA: enzyme‐linked immunosorbent assay
GSDMD‐N: N‐terminus of Gasdermin D
HRP: horseradish peroxidase
IL‐1β: interleuckin‐1beta
LDH: lactate dehydrogenase
LPS: lipopolysaccharide
NLRP3: NLR family pyrin domain containing 3
PAMPs: pathogen‐associated molecular patterns
PI: propidium iodide
qPCR: quantitative real‐time polymerase chain reaction
RIPA: radioimmunoprecipitation assay
SA‐AKI: sepsis‐associated acute kidney injury
shRNA: small hairpin RNA
TNF‐α: tumor necrosis factor‐alpha
USP14: ubiquitin‐specific protease 14

## 1 Introduction

Sepsis is a severe condition characterized by excessive inflammatory reaction, commonly triggered by infection, which can lead to a life‐threatening situation [1, 2]. Acute kidney injury (AKI) represents a prevalent and potentially lethal complication associated with sepsis, accounting for approximately 40% of sepsis cases [3]. Compared to non‐infectious AKI, sepsis‐associated AKI (SA‐AKI) has more complex etiologies, results in more severe renal dysfunction, and imposes a greater burden on patients [4]. Nevertheless, the precise pathophysiological mechanisms that underlie sepsis‐induced AKI remain incompletely elucidated, thereby constraining the advancement of effective therapeutic interventions. Consequently, a thorough investigation into the mechanisms implicated in the initiation and progression of SA‐AKI is imperative. Such exploration could facilitate the development of novel therapeutic targets and pave the way for innovative treatment approaches.

Numerous studies have demonstrated that a large amount of inflammatory factors are released during the pathogenesis of sepsis‐induced AKI, leading to inflammation and the injury of renal tubular epithelial cells, while pyroptosis plays a pivotal role in this process [5]. Pyroptosis, a type of programmed cell death initiated by inflammatory signals, has been increasingly recognized in a variety of diseases especially those associated to inflammation. This process crucially contributes to renal impairment, highlighting its importance in the pathophysiology of kidney disease [6, 7]. Moreover, programmed cell death of renal tubular epithelial cells, including pyroptosis, has been validated as one of the mediators of sepsis‐induced AKI [8]. Therefore, investigating strategies aimed at targeting pyroptosis could emerge as a promising therapeutic approach for SA‐AKI.

Ubiquitin‐specific protease 14 (USP14) functions as a deubiquitinating enzyme (DUB) correlated to the proteasome. It is responsible for the removal of ubiquitin from the proteasome and the targeting of proteins that have been ubiquitinated. USP14 plays a pivotal role in acute renal injury and in inflammation triggered by sepsis, underscoring its significance in the pathophysiology of these conditions [9, 10]. The deubiquitinating activity of USP14 is crucial for the activation of the inflammasome, while inhibition of this activity can effectively prevent the release of interleukin‐1beta (IL‐1β) and the activation of Caspase1 [11]. Inhibition of USP14 can mitigate the effects of sepsis, which is induced by cecal ligation and puncture (CLP), and attenuate lipopolysaccharide (LPS)‐triggered inflammatory responses *in vivo* and *in vitro* [10]. In renal injury scenarios, silencing USP14 has been shown to reduce ischemia‐reperfusion‐induced kidney damage [12]. Nonetheless, the precise roles and mechanisms by which USP14 contributes to SA‐AKI remain unclear.

Substantial evidence indicates that NLR family pyrin domain containing 3 (NLRP3) inflammasome is pivotal in the pathogenesis and progression of SA‐AKI. Its activation is inevitable for the maturation and release of pro‐inflammatory cytokines, as well as for initiating pyroptosis, thereby contributing to the inflammatory response and cell death observed in SA‐AKI [13, 14]. Deubiquitinating enzymes are known to influence the progression of inflammatory diseases through the post‐translational modification of NLRP3 [15, 16, 17]. In particular, studies focusing on pyroptosis have shown an association between USP14 and NLRP3 [16]. However, the existing literature has not yet specifically described such an interaction in the context of AKI. Consequently, this study is aimed to elucidate the mechanisms by which USP14 modulates NLRP3 and pyroptosis during SA‐AKI.

## 2 Materials and Methods

### 2.1 Experimental Animals

Twenty‐four healthy, six to eight‐week‐old male C57BL/6 mice were obtained from Vital River (Beijing, China). The mice underwent a 72‐h acclimatization period in a temperature‐regulated environment (22 ± 2°C), with continuous access to sterilized food and water prior to the start of the experimental protocols. All experimental protocols were strictly followed in accordance with the ethical guidelines for the welfare and utilization of laboratory animals as prescribed by the Animal Ethics Committee of the Affiliated Yueqing Hospital of Wenzhou Medical University (YQYY20250013).

### 2.2 Animal Models and Treatments

The CLP operation was applied for inducing a sepsis animal model. Mice were fasted for 12 h before surgery and then were anesthesized 50 mg/kg of sodium pentobarbital intraperitoneal injection. The abdominal region was carefully shaved and disinfected to ensure aseptic conditions. Subsequently, a midline incision was performed to exposed the cecum for further experimental procedures. The cecum was ligated below the ileocecal valve and punctured. The cecum was then gently squeezed to extrude a little amount of feces, and the abdomen was closed. Mice in control group underwent the same procedure except ligation or puncture. Postoperatively, mice were subcutaneously injected with warm saline (1 mL) to compensate for fluid loss. Mice were subsequently divided into different groups (*n* = 6 per group) radomly, including Control group, CLP group, CLP plus Ad‐sh‐NC (negative control adenovirus) group, and CLP plus Ad‐sh‐USP14 (USP14‐targeting adenovirus; 5′‐CTACCTGCTTACTTAACTATT‐3′) group. Adenoviruses were administered via tail vein injection at a dose of 1 × 10^9^ plaque‐forming units, 2 weeks prior to CLP surgery. The sample size (six mice per group) was predetermined based on published literatures and previous lab experience, without statistical methods. No samples were excluded from future analysis.

### 2.3 Renal Function Assessment

Blood samples, approximately 0.5 mL in volume, were extracted from the retro‐orbital plexus of anesthetized animals 24 h following CLP. The samples were allowed to clot at room temperature for 30 min prior to centrifugation at 3000 rpm for 15 min to obtain the serum. Serum creatinine and blood urea nitrogen (BUN) levels were measured using a biochemical analyzer (Hitachi, Japan). Concurrently, urine was collected over a 24‐h period in metabolic cages, with the urine volume being meticulously recorded.

### 2.4 Histological Analysis

At 24 h following the CLP procedure, the mice were euthanized under anesthesia with 5% isoflurane, and their kidneys were collected. The harvested kidneys were subsequently immersed in 4% paraformaldehyde for fixation. After that, the kidneys were transferred to 70% ethanol for dehydration, which facilitated the paraffin embedding process. Finally, thin sections of the embedded tissues were prepared using a microtome (Leica, Germany). Tissue sections were first deparaffinized using xylene and subsequently rehydrated through an ethanol gradient. Afterward, the sections were stained with hematoxylin for 5 min, followed by a rinse in tap water. Subsequently, eosin staining was applied for 2 min. Then, slides were dehydrated, cleared, and mounted with coverslips with a synthetic resin. Sections were examined using a light microscope (Olympus, Japan). Renal injury was assessed in a blinded manner using a semi‐quantitative scoring system based on the percentage of damaged tubules: 0 indicates no damage; 1 indicates damage of less than 25%; 2 indicates damage ranging from 25% to 50%; 3 indicates damage ranging from 50% to 75%; and 4 indicates damage exceeding 75%.

### 2.5 Cell Culture and Treatments

Human renal proximal tubular epithelial cells (HK‐2) were obtained from Wuhan Pricella Biotechnology Co. Ltd. (CL‐0109, China). Cells were cultured in the specific culture medium (CM‐0109, Pricella) at 37°C in a humidified incubator with 5% CO_2_ in the air. The cells were exposed to 1 μg/mL lipopolysaccharide (LPS; Sigma‐Aldrich, USA) in 6‐well or 12‐well plates for 24 h to simulate a sepsis‐like condition [18]. For the inhibition of NLRP3, cells were treated with 10 µM of MCC950 (MedChemExpress, USA).

### 2.6 Cell Transfection

Cells were transfected with small hairpin RNA (shRNA) targeting USP14 (sh‐USP14; 5′‐CGCAGAGTTGAAATAATGGAA‐3′; GenePharma, Shanghai, China) or non‐targeting control shRNA using Lipofectamine 3000 agent (Invitrogen, USA) following the manufacturer's guidance. In brief, HK‐2 cells were initially plated in 6‐well plates at a density of 2 × 10^5^ cells per well and cultured until they achieved 70%‐80% confluence before transfection. The shRNA constructs were diluted in Opti‐MEM (Invitrogen), while Lipofectamine 3000 was diluted separately in Opti‐MEM. After a 5‐min incubation at room temperature, the diluted shRNA and Lipofectamine 3000 were co‐incubated for an additional 20 min to form complexes. The shRNA‐Lipofectamine complexes were then added dropwise to the cells and incubated for 6 h. Following the initial incubation period, the used medium was substituted with fresh, complete growth medium. Next, the cells were cultured for an additional 48 h prior to the commencement of the experimental procedures.

### 2.7 Quantitative Real‐Time Polymerase Chain Reaction (qPCR) Assay

RNA was extracted using the TRIzol reagent (Invitrogen, USA), adhering to the protocol specified by the manufacturer. Initially, the cells or tissues were lysed within the TRIzol solution, and chloroform was introduced to promote phase separation. Post‐centrifugation, the aqueous layer was carefully extracted, and RNA precipitation was achieved through the use of isopropanol. The obtained pellets were rinsed with 75% ethanol and then reconstituted in nuclease‐free water. Following this, reverse transcription was conducted, and qPCR was carried out utilizing the SYBR Green Kit (Takara, Japan) on a Real‐Time PCR System (Takara). The corresponding primer sequences are listed in Table 1. The relative expression of each gene was calculated using the 2^–ΔΔCt^ method and normalized to GAPDH expression. GAPDH was used as the internal reference gene, and its stable expression across all experimental groups was validated by the low variability of cycle threshold values (standard deviation < 0.5; *p* > 0.05).

**Table 1:** Primers used in this study.

Gene	Sequence (5'–3')
USP14 (mouse)	CCTCTGCTAAAACTGTCTTCGTAG
	GGCATCTTTGAGTTCAGGCACAG
USP14 (human)	GGGAAATGGCTTCAGCGCAGTA
	CACCTTTCTCGGCAAACTGTGG
IL‐1β (mouse)	TGGACCTTCCAGGATGAGGACA
	GTTCATCTCGGAGCCTGTAGTG
IL‐1β (human)	CCACAGACCTTCCAGGAGAATG
	GTGCAGTTCAGTGATCGTACAGG
IL‐18 (mouse)	GACAGCCTGTGTTCGAGGATATG
	TGTTCTTACAGGAGAGGGTAGAC
IL‐18 (human)	GATAGCCAGCCTAGAGGTATGG
	CCTTGATGTTATCAGGAGGATTCA
TNF‐α (mouse)	GGTGCCTATGTCTCAGCCTCTT
	GCCATAGAACTGATGAGAGGGAG
TNF‐α (human)	CTCTTCTGCCTGCTGCACTTTG
	ATGGGCTACAGGCTTGTCACTC
GAPDH (mouse)	CATCACTGCCACCCAGAAGACTG
	ATGCCAGTGAGCTTCCCGTTCAG
GAPDH (human)	GTCTCCTCTGACTTCAACAGCG
	ACCACCCTGTTGCTGTAGCCAA

### 2.8 Western Blot

Protein lysates were obtained using radioimmunoprecipitation assay (RIPA) buffer (Byotime, Shanghai, China), and the protein concentration was quantified with the bicinchoninic acid (BCA) Protein Assay Kit (Transgen, Beijing, China). Protein samples were then resolved using sodium dodecyl sulfate‐polyacrylamide gel electrophoresis on 10% polyacrylamide gels. Following electrophoresis, the proteins were transferred to polyvinylidene fluoride membranes (Millipore, USA) and incubated in a blocking solution of 5% non‐fat milk for 1 h at room temperature. Membranes were then exposed to primary antibodies overnight at 4°C. Primary antibodies used were rabbit anti‐USP14 (1:5000, Cat No. 14517‐1‐AP, Proteintech, Wuhan, China), rabbit anti‐NLRP3 (1:1000, Cat No. 30109‐1‐AP, Proteintech), rabbit anti‐N‐terminus of Gasdermin D (GSDMD‐N; 1:1000, Cat No. 36425/10137, Cell Signaling Technology), rabbit anti‐cleaved Caspase1 (1:1000, Cat No. 89332/4199, Cell Signaling Technology), and rabbit anti‐β‐actin (1:1000, Cat No. 66009‐1‐Ig, Proteintech). Membranes were then incubated with horseradish peroxidase (HRP)‐conjugated secondary antibodies (1:1000, Cat No. SA00001‐2, Proteintech) for 1 h. Bands were observed using an Electrochemiluminescence Detection System (Bio‐Rad, USA). Band intensities were quantified using imageJ analysis software.

### 2.9 Enzyme‐Linked Immunosorbent Assay (ELISA)

The levels of IL‐1β, interleuckin (IL)‐18, and tumor necrosis factor‐alpha (TNF‐α) in serum and cell culture supernatants were determined via ELISA using commercial kits (IL‐1β: E‐EL‐M0037, E‐EL‐H0149; IL‐18: E‐EL‐M0730, E‐EL‐H0253; TNF‐α: E‐EL‐M3063, E‐EL‐H0109) following the manufacturer's instructions (Elabscience, Wuhan, China). Absorbance was read at 450 nm using a microplate reader (Bio‐Rad, USA). Concentrations were calculated using a standard curve generated with known concentrations of recombinant cytokines. In addition, Lactate Dehydrogenase (LDH) Cytotoxicity Assay Kit (C0017, Beyotime) was used to assess LDH release level from HK‐2 cells following the instructions.

### 2.10 Cell Viability Assay

Cell counting kit‐8 (CCK‐8) assay was employed for assessing the cell viability. HK‐2 cells were seeded in 96‐well plates. The cell density was 5 × 10^3^ cells per well. After the corresponding treatments, 10 µL of CCK‐8 solution (Donjido, Japan) was added into each well and then incubated for 2 h at 37°C in a CO_2_ incubator. The 450 nm absorbance was read using a microplate reader (Bio‐Rad).

### 2.11 Flow Cytometry

FLICA 660 Caspase‐1 Assay Kit (ICT‐9122, ImmunoChemistry) was used to assess pyroptosis of HK‐2 cells. At the end of each treatment, cells were trypsinized, washed with PBS, and stained with FLICA 660‐YVAD‐FMK and propidium iodide (PI). After washing, cells were analyzed by flow cytometry (BD Biosciences). Pyroptotic cells were identified as double positive for Caspase1 and PI.

### 2.12 Co‐Immunoprecipitation (Co‐IP) and Ubiquitination Assay

Protein lysates were prepared in RIPA buffer, and clarified lysates were incubated with rabbit anti‐USP14 antibody (14517‐1‐AP, Proteintech), rabbit anti‐NLRP3 antibody (30109‐1‐AP, Proteintech), or normal rabbit IgG (Santa Cruz Biotechnology, USA) overnight at 4°C. Agarose beads were added and incubated for an additional 2 h at 4°C. Following this step, beads were washed triple times by RIPA buffer and resuspended in 2× Laemmli sample buffer. The immune complexes were analyzed by western blot for the presence of NLRP3, USP14, or ubiquitin (1:1000, ab134953, Abcam).

### 2.13 Statistical Analysis

Data were expressed as mean ± standard deviation. Graphs and statistical evaluations were conducted using GraphPad Prism software (version 8.0.1; GraphPad, La Jolla, CA, USA). Prior to parametric analyses, normality was checked via the Shapiro‐Wilk test, and homogeneity of variances via the Brown‐Forsythe test. One‐way analysis of variance (ANOVA) followed by Tukey's post hoc test was utilized for comparisons among multiple groups, while an unpaired Student's *t*‐test was used for comparing two groups. A *p*‐value of less than 0.05 was deemed statistically significant. This study has been reported in accordance with the ARRIVE guidelines 2.0: author checklist.

## 3 Results

### 3.1 Knockdown of USP14 Alleviates Sepsis‐Induced AKI

The expression of USP14 was detected in the kidney tissues after different treatments. As shown in Figure 1A,B, the protein and mRNA levels of USP14 were significantly upregulated in the renal tissues of mice with CLP‐induced sepsis. Knocking down USP14 effectively decreased the levels of USP14 mRNA and protein in the renal tissues. Moreover, the septic mice exhibited substantial elevation in serum creatinine and BUN levels, accompanied by a notable reduction in 24‐h urine output. Inhibition of USP14 decreased the serum creatinine and BUN levels and increased the 24‐h urine volume (Figure 1C). Histological examination of renal tissues from the CLP group revealed several features, including swelling of renal tubular epithelial cells, vacuolar degeneration, and dilation of the renal tubular lumen, resulting in an elevated score for renal tubular injury. The knockdown of USP14 alleviated renal tubular injury and reduced the injury score (Figure 1D).

**Figure 1:**
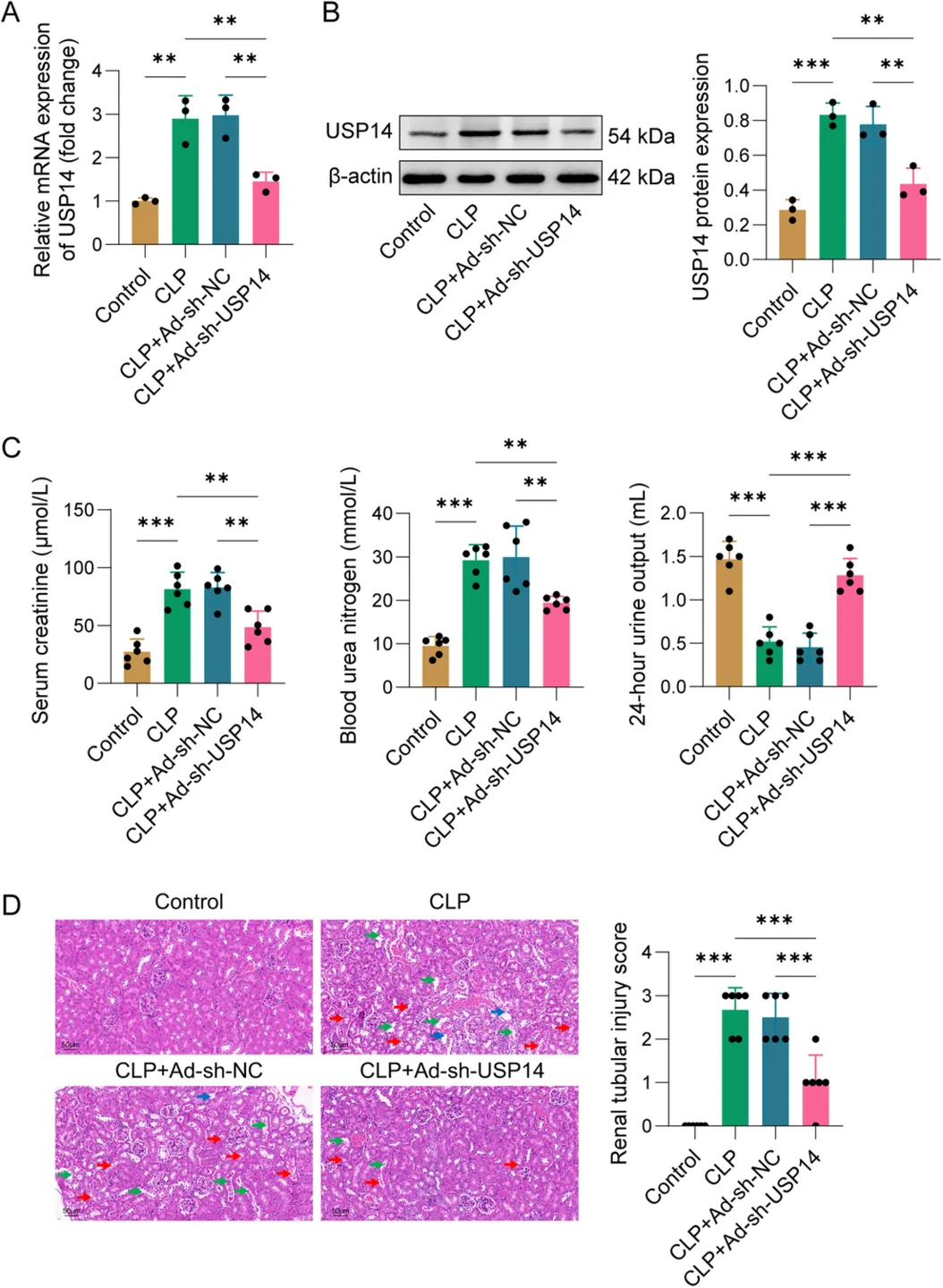
Knockdown of USP14 alleviates sepsis‐induced AKI. (A) USP14 mRNA expression in kidney tissues were detected by qPCR. (B) USP14 protein levels in kidney tissues were detected by Western blot. *n* = 3. CLP stimulation upregulated USP14 (**p < 0.01 or ***p < 0.001 vs. Control), while USP14 knockdown significantly reduced both mRNA and protein levels compared with the Ad‐sh‐NC group (**p < 0.01). (C) Serum creatinine and BUN levels were detected and 24‐h urine volume was recorded. (D) Kidney injury was evaluated by H&E staining. Blue arrow indicates vacuolar degeneration; red arrow indicates swelling of renal tubular epithelial cells; green arrow indicates dilation of the renal tubular lumen. *n* = 6. **p < 0.01, ***p < 0.001.

### 3.2 Knockdown of USP14 Inhibits Pyroptosis in Septic AKI

Several studies indicated that silencing USP14 can inhibit pyroptosis in multiple diseases [11, 19], thus we considered a similar phenomenon could be observed in SA‐AKI. Relative to the control group, the levels of IL‐1β, IL‐18, and TNF‐α mRNA were markedly increased in the kidney tissues of mice from the CLP group. Knocking down USP14 suppressed the upregulation of these inflammatory genes mediated by CLP (Figure 2A). Similarly, the serum levels of IL‐1β, IL‐18, and TNF‐α in mice from the CLP group were notably increased compared to the control group. Knockdown of USP14 partially reversed the elevation of IL‐1β, IL‐18, and TNF‐α levels induced by CLP (Figure 2B). Furthermore, CLP induction increased the levels of NLRP3, GSDMD‐N, and cleaved Caspase1 proteins in the kidney tissues of mice. The silencing of USP14 inhibited the upregulation of NLRP3, GSDMD‐N, and cleaved Caspase1 proteins induced by CLP in the kidney tissues (Figure 2C). These findings suggest that knockdown of USP14 reduces pyroptosis in mice with septic AKI.

**Figure 2:**
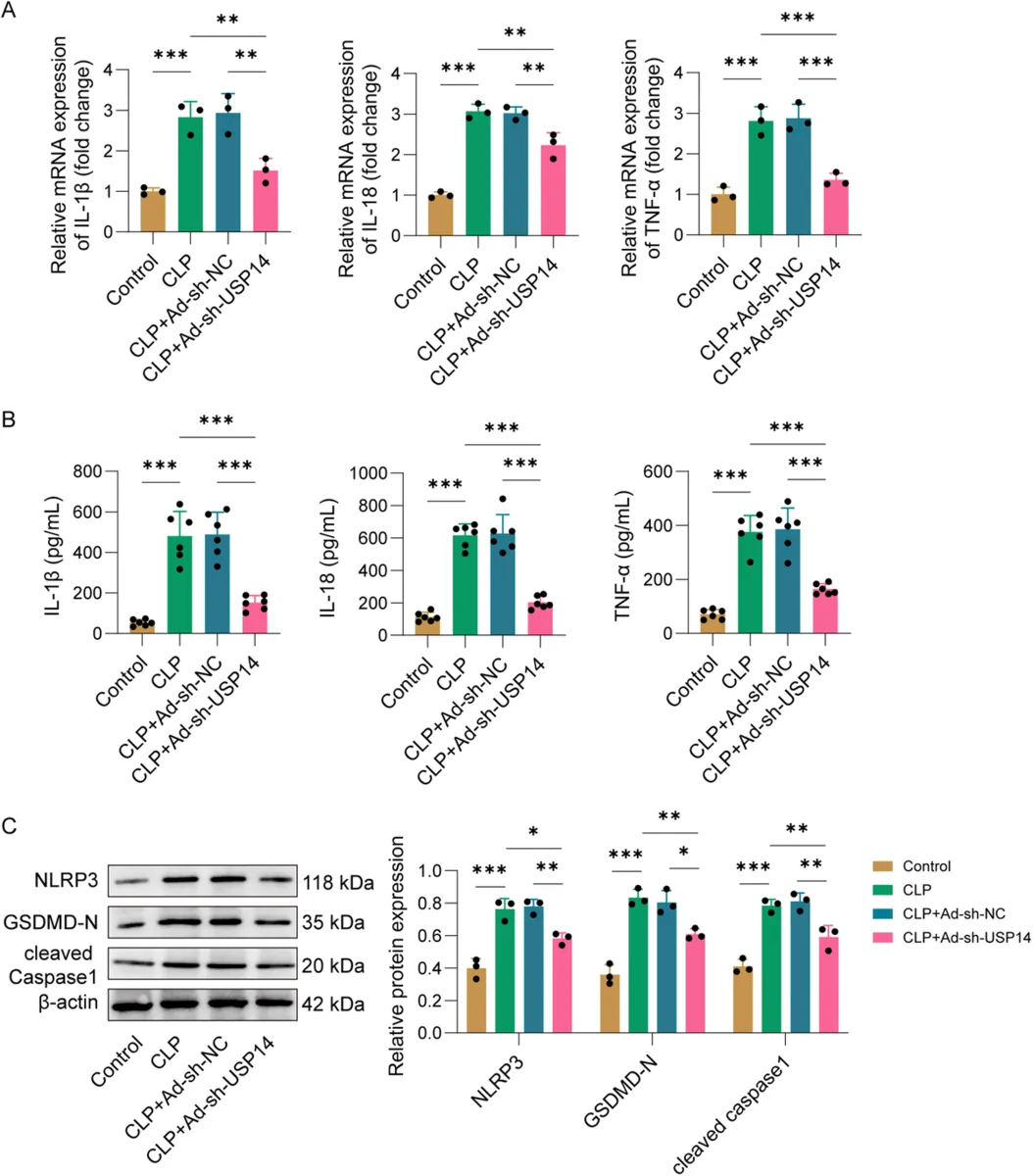
Knockdown of USP14 inhibits pyroptosis in septic AKI. (A) IL‐1β, IL‐18, and TNF‐α mRNA expression in kidney tissues were detected by qPCR. *n* = 3. (B) Serum IL‐1β, IL‐18, and TNF‐α levels were detected using ELISA kits. *n* = 6. (C) Protein levels of NLRP3, GSDMD‐N, and cleaved Caspase1 in kidney tissues were detected by Western blot. *n* = 3. *p < 0.05, **p < 0.01, ***p < 0.001.

### 3.3 Silencing USP14 Inhibits LPS‐Induced Inflammation in HK‐2 Cells

LPS significantly upregulated both USP14 mRNA and protein levels, whereas sh‐USP14 markedly reduced the LPS‐induced increase in USP14 mRNA and protein levels (Figure 3A,B). Furthermore, LPS markedly enhanced the mRNA expression levels of IL‐1β, IL‐18, and TNF‐α in cells, while USP14 knockdown inhibited these gene expression (Figure 3C). Similarly, LPS significantly elevated the levels of IL‐1β, IL‐18, and TNF‐α in the cell supernatants, and knockdown of USP14 reduced the production of these inflammatory factors (Figure 3D). Collectively, these findings indicate that USP14 knockdown can inhibit LPS‐induced inflammation in renal tubular epithelial cells.

**Figure 3:**
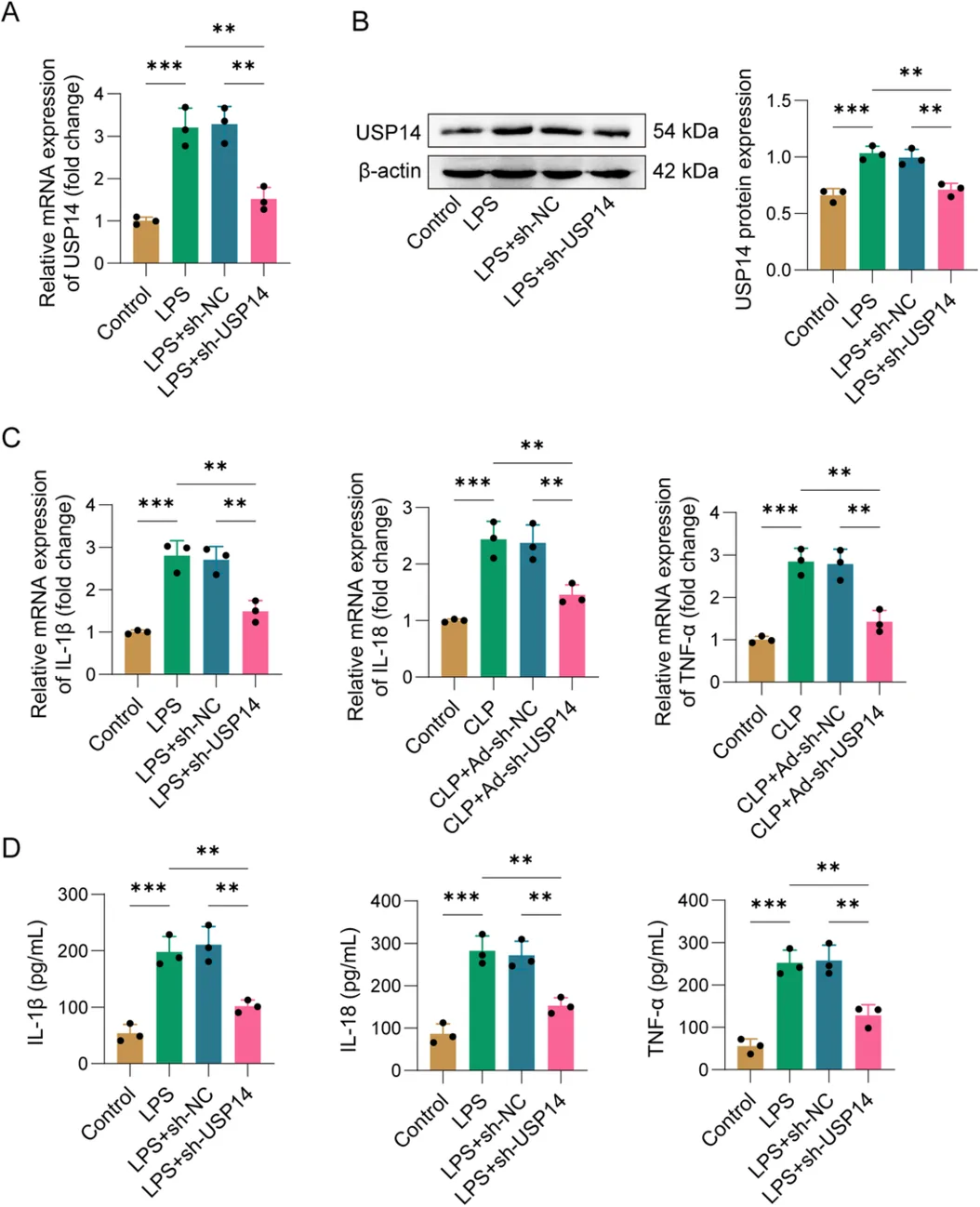
Silencing USP14 inhibits LPS‐induced inflammation in renal tubular epithelial cells. (A) USP14 mRNA expression was detected by qPCR. (B) USP14 protein levels were detected by Western blot. (C) The mRNA expression of IL‐1β, IL‐18, and TNF‐α was detected by qPCR. (D) Levels of IL‐1β, IL‐18, and TNF‐α were detected by ELISA. *n* = 3. **p < 0.01, ***p < 0.001.

### 3.4 USP14 Deubiquitinates NLRP3

LPS significantly upregulated NLRP3 protein levels, while knockdown of USP14 inhibited NLRP3 expression (Figure 4A). Moreover, NLRP3 was detectable in the IP complex of the USP14 group, indicating that USP14 bound to NLRP3 (Figure 4B). The ubiquitination detection results showed that LPS induced a reduction in NLRP3 ubiquitination level, whereas USP14 knockdown increased LPS‐induced NLRP3 ubiquitination level in cells (Figure 4C). These results suggest that USP14 knockdown can enhance NLRP3 ubiquitination and reduce NLRP3 expression.

**Figure 4:**
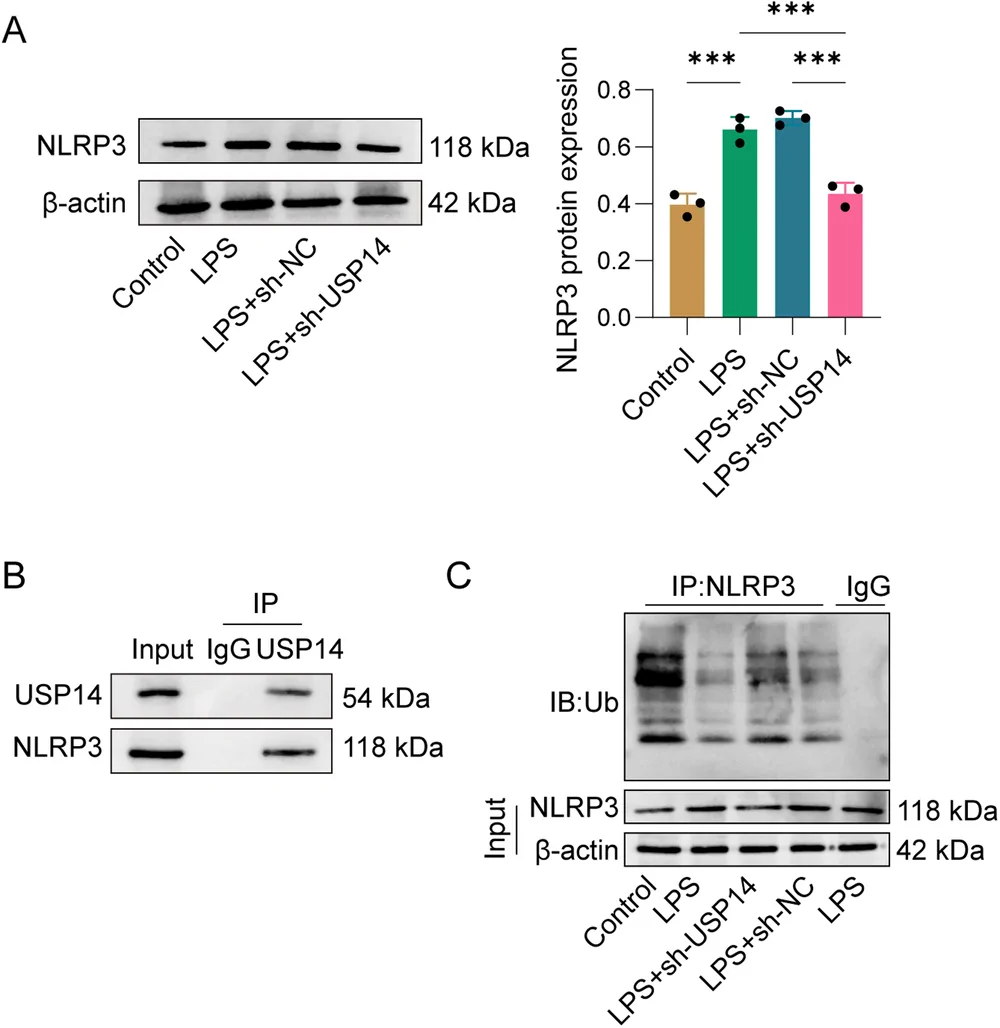
USP14 deubiquitinates NLRP3. (A) NLRP3 expression was detected by Western blot. (B) The interaction between USP14 and NLRP3 was validated by Co‐IP. (C) The impact of USP14 on the ubiquitination levels of NLRP3 was detected by IP/western blot. Input, total cell lysates prior to immunoprecipitation, showing baseline expression of the indicated proteins and serving as a loading control across samples. *n* = 3. ***p < 0.001.

### 3.5 USP14 Knockdown Inhibits NLRP3‐mediated Pyroptosis in LPS‐Induced HK‐2 Cells

HK‐2 cell viability was decreased under LPS stimulation, while the inhibition of NLRP3 or knockdown of USP14 can increase LPS‐induced cell viability (Figure 5A). Additionally, LPS induced an increase in LDH level, whereas the inhibition of NLRP3 or knockdown of USP14 can reduce LPS‐induced LDH level (Figure 5B). Furthermore, the inhibition of NLRP3 or knockdown of USP14 downregulated the LPS‐induced levels of GSDMD‐N and cleaved Caspase1 proteins in HK‐2 cells (Figure 5C). LPS also induced a significant increase in Caspase1/PI positive cells, while the inhibition of NLRP3 or knockdown of USP14 reduced the number of LPS‐induced Caspase1/PI positive cells (Figure 5D). To further verify whether USP14 knockdown inhibits pyroptosis specifically through NLRP3 downregulation, we performed rescue experiments in LPS‐stimulated HK‐2 cells. As shown in Figure S1, compared with LPS treatment, knockdown of USP14 or NLRP3 alone significantly reduced the levels of IL‐1β, IL‐18, LDH release, and the percentage of Caspase‐1/PI‐positive cells. Notably, simultaneous knockdown of USP14 and NLRP3 did not result in a further reduction of these pyroptosis markers compared with USP14 knockdown alone, consistent with NLRP3 acting downstream of USP14. Conversely, overexpression of NLRP3 in USP14‐knockdown cells significantly reversed the USP14 knockdown‐mediated suppression of these pyroptosis markers. Collectively, these results suggest that the knockdown of USP14 inhibits LPS‐induced pyroptosis in HK‐2 cells by downregulating NLRP3.

**Figure 5:**
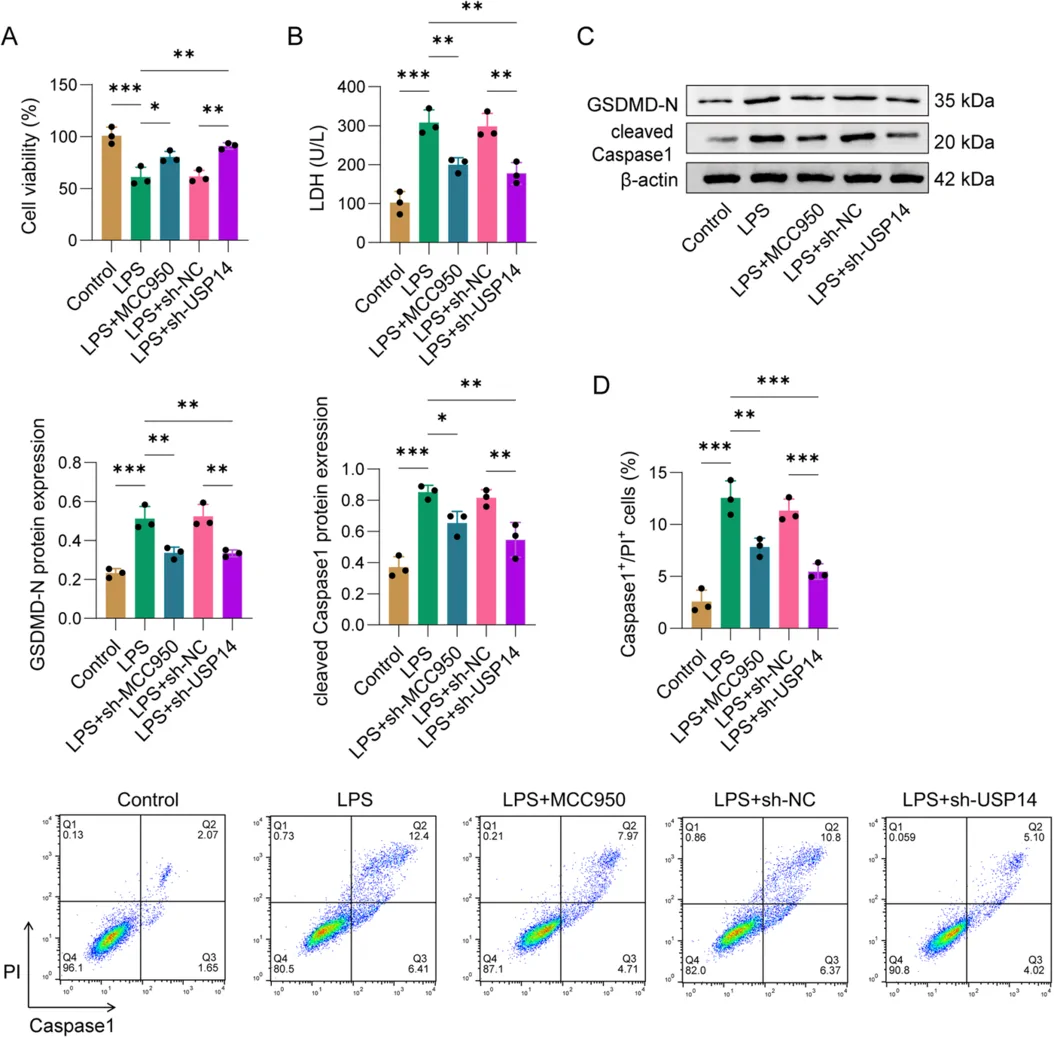
USP14 knockdown inhibits NLRP3‐mediated pyroptosis in LPS‐induced renal tubular epithelial cells. (A) Cell viability was measured by CCK‐8 assay. (B) LDH release level was detected using a reagent kit. (C) Protein levels of GSDMD‐N and cleaved Caspase1 were detected by Western blot. (D) Pyroptosis was evaluated by flow cytometry to detect Caspase1/PI‐positive cells. *n *= 3. *p < 0.05, **p < 0.01, ***p < 0.001.

## 4 Discussion

Sepsis is a severe systemic inflammatory response to infection characterized by an overproduction of various inflammatory mediators, leading to extensive cellular and tissue damage [20]. Among the organs, the kidneys are particularly susceptible to the progression of sepsis [21]. The release of inflammatory factors that induce pyroptosis in cells is a critical aspect of the pathogenesis of sepsis. For instance, Ye et al. reported that pyroptosis mediated by caspase‐11 in tubular cells is a critical event in SA‐AKI, thus inhibiting pyroptosis in cells may be a crucial event in the treatment of SA‐AKI [22]. Our work suggests that USP14 plays a crucial role in SA‐AKI. Knockdown of USP14 significantly reduces the NLRP3 inflammasome activation, thereby mitigating pyroptosis in AKI. These findings support the idea that USP14 could serve as a promising therapeutic target for mitigating sepsis‐induced AKI.

USP14 belongs to the family of ubiquitin‐specific proteases and is essential for regulating the activity of deubiquitinating enzymes, influencing the degradation of proteins as well as the stability and function of specific target proteins [16, 23]. The process of ubiquitination is essential for regulating various cellular activities. Recently, USP14 has emerged as a promising therapeutic target for a range of conditions, including inflammatory diseases [24, 25]. USP14 is implicated in inflammatory regulation in chondrocytes by promoting IκBα deubiquitination and degradation [26]. In our research, USP14 was found markedly upregulated in the renal tissues of septic AKI mice, while its downregulation effectively decreased the levels of several inflammation factors including IL‐1β, IL‐18, and TNF‐α. Moreover, USP14 is upregulated in hypoxia/reoxygenation (H/R)‐induced HK‐2 cells and mouse models with ischemic kidney injury [9]. The knockdown of USP14 in ischemia/reperfusion‐induced AKI can mitigate renal epithelial cell inflammation and apoptosis by interacting and deubiquitinating its downstream target gene [12]. Consistently, we found that the silencing of USP14 could reduce septic kidney injury in mice.

The NLRP3 inflammasome and pyroptosis are intricately connected, with pyroptosis serving as a programmed cell death pathway regulated by Caspase1, playing a crucial role in anti‐infection immunity and inflammatory responses [15, 27]. The NLRP3 inflammasome is composed of NLRP3, apoptosis‐associated speck‐like protein containing a CARD (ASC), and pro‐Caspase1, which sense pathogen‐associated molecular patterns (PAMPs) and damage‐associated molecular patterns (DAMPs) [28, 29]. Upon activation, NLRP3 undergoes oligomerization, recruiting ASC and pro‐Caspase1. Once activated, pro‐Caspase1 cleaves pro‐IL‐1β and pro‐IL‐18 to generate active cytokines [30]. Additionally, it cleaves GSDMD to form membrane pores, resulting in cell swelling, lysis, and the release of pro‐inflammatory cytokines [31]. This process is not only critical for host defense but also amplifies local and systemic inflammatory responses by releasing pathogens and cytokines [32]. As expected, MCC950 (a specific inhibitor of NLRP3) inhibits LPS‐induced production of IL‐18 and IL‐1β and pyroptosis in HK‐2 cells [14], which is consistent with our results. The inhibition of NLRP3‐mediated pyroptosis protects against kidney damage and inflammation in LPS‐stimulated renal tubular cells and septic mice [33]. Moreover, NLRP3 can be deubiquitinated in inflammation‐associated diseases [34]. In this study, we proved that USP14 deubiquitinates NLRP3 and thus enhances its stability. Moreover, knockdown of USP14 markedly repressed the LPS‐induced pyroptosis. The reduction in GSDMD‐N levels upon USP14 knockdown is mechanistically and pathophysiologically significant. As the N‐terminal pore‐forming fragment cleaved by active Caspase‐1, GSDMD‐N oligomerizes on the plasma membrane, compromising cell integrity, driving the release of pro‐inflammatory cytokines (e.g., IL‐1β and IL‐18) and intracellular damage‐associated molecules (e.g., LDH), thereby serving as the final executioner of pyroptosis [16, 35]. In our study, USP14 knockdown markedly decreased GSDMD‐N generation in both CLP‐induced septic mice and LPS‐stimulated HK‐2 cells, correlating with the attenuated inflammatory responses observed. Notably, this suppressive effect was largely reversed by NLRP3 overexpression in USP14‐silenced cells, confirming that USP14 regulates GSDMD‐N maturation indirectly through the upstream NLRP3/Caspase‐1 axis, rather than via direct deubiquitination of GSDMD itself. Given that GSDMD‐N‐driven pore formation exacerbates the systemic inflammatory cascade and organ injury in sepsis, these results position USP14 as a potential upstream therapeutic target to mitigate sepsis‐associated pyroptotic damage.

## 5 Limitations

Several limitations of this study should be acknowledged. First, although we have established a pathogenic role for USP14 in SA‐AKI, the specific cellular compartments or tubular segments in which USP14 exerts its effects remain to be further defined. Second, our in vivo experiments were performed exclusively in a murine CLP model, and the translational relevance of our findings to human SA‐AKI awaits validation in clinical samples. Third, we only evaluated the acute phase (24 h post‐CLP) of renal injury, leaving the long‐term consequences of USP14 inhibition—such as effects on renal fibrosis, functional recovery, and overall survival—unexplored. Addressing these questions in future studies will be essential to fully assess the therapeutic potential of targeting USP14 in sepsis.

## 6 Conclusions

In conclusion (Figure 6), the results presented above demonstrate that USP14 deubiquitinates NLRP3, thereby enhancing pyroptosis and facilitating SA‐AKI. Our study identifies USP14 as a potential therapeutic target for SA‐AKI. Future studies should evaluate selective USP14 inhibitors in therapeutic regimens post‐sepsis onset to mimic clinical practice. Whether USP14 inhibition confers protection in other sepsis‐affected organs warrants investigation. Additionally, the specific lysine residues of NLRP3 targeted by USP14 remain to be mapped. Finally, validation of USP14/NLRP3 expression in human SA‐AKI kidney samples will be critical to strengthen clinical translatability.

**Figure 6:**
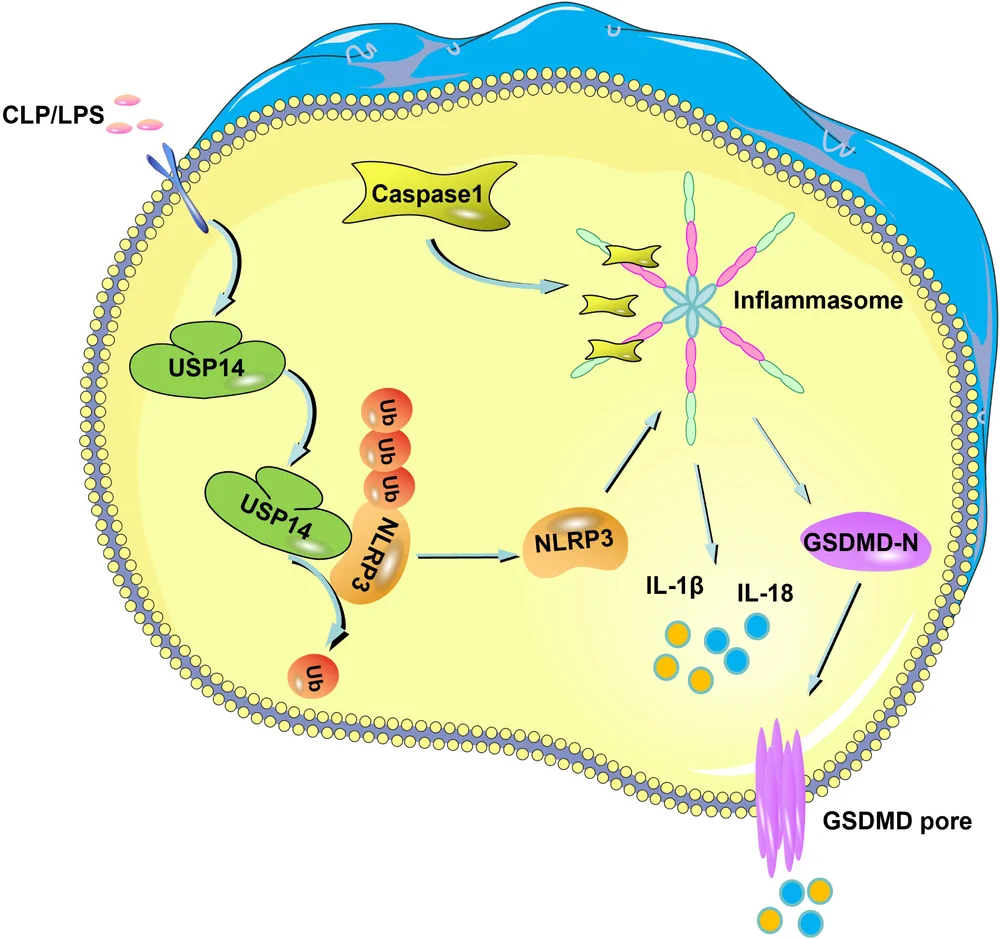
Schematic diagram illustrating the mechanism by which USP14 regulates pyroptosis in SA‐AKI. In response to LPS or CLP, upregulation of USP14 can induce activation of pyroptosis. USP14 promotes NLRP3 expression by mediating NLRP3 deubiquitination, thereby inducing the higher order assembly of NLRP3 inflammasome. Activated Caspase1 promotes the maturation of the cytokines IL‐1β and IL‐18 and the formation of GSDMD pore.

## Author Contributions

Jini Zheng, Wanglong Chen and Yangyang Ye guaranteed the integrity of the entire study and designed the study and literature research. Jini Zheng, Wanglong Chen and Ming Zheng defined the intellectual content. Dan Zhu and Lei Zheng performed experiment and collected the data. Qian Zhao and Xiaoqiong Huang analyzed the data. Jini Zheng, Dan Zhu, and Ming Zheng wrote the main manuscript and prepared figures. All authors reviewed the manuscript.

## Ethics Statement

All experimental protocols were strictly adhered to, following the ethical guidelines for the welfare and utilization of laboratory animals as prescribed by the Animal Ethics Committee of Affiliated Yueqing Hospital of Wenzhou Medical University (YQYY20250013).

## Conflicts of Interest

The authors declare no conflicts of interest.

## Supporting information


**Figure S1:** USP14 knockdown inhibits pyroptosis through NLRP3. (A) IL‐1β levels in cell culture supernatants measured by ELISA. (B) IL‐18 levels in cell culture supernatants measured by ELISA. (C) LDH release into the culture supernatant measured by cytotoxicity assay. (D) Percentage of Caspase‐1/PI‐positive cells (pyroptotic cells) determined by flow cytometry. n = 3. **p < 0.01, ***p < 0.001.

Supporting File

## Data Availability

The data that support the findings of this study are available from the corresponding author upon reasonable request.
